# Synthesis, characterization and evaluation of experimental dental composite resin modified by grapefruit seed extract-mediated TiO₂ nanoparticles: green approach

**DOI:** 10.1007/s10266-025-01058-9

**Published:** 2025-02-17

**Authors:** Dina Ezzat, Mai Samy Sheta, El-Refaie Kenawy, Mohammed A. Eid, Hend Elkafrawy

**Affiliations:** 1 Dental Biomaterials Department, Faculty of Dentistry, Tanta University, El-Geish Street, Tanta, 31511 Egypt; 2 Chemistry Department, Faculty of Science, Tanta University, Tanta, Egypt; 3 Botany and Microbiology Department, Faculty of Science, Tanta University, Tanta, Egypt

**Keywords:** Green TiO_2_NPs, Grapefruit seed extract (GSE), Green nanotechnology, Antibacterial composite resin, Post-gel polymerization shrinkage

## Abstract

Dental composite resins often face challenges related to secondary caries, polymerization shrinkage and fracture failure. This study aimed to synthesize an experimental composite resin modified with grapefruit seed extract-mediated titanium dioxide nanoparticles (GSE-TiO₂NPs) and evaluate its antibacterial activity and mechanical and physical properties. Green synthesis of TiO₂ nanoparticles was conducted utilizing GSE. The chemical profile of GSE was identified through gas chromatography-mass spectrometry. The as-prepared nanoparticles were incorporated into experimental composites at concentrations of 10 wt.% (10 wt.% GSE-TiO₂NPs group) and 20 wt.% (20 wt.% GSE-TiO₂NPs group), with an unmodified composite as the control group. Scanning electron microscopy (SEM) and transmission electron microscopy (TEM) confirmed the morphology and size of synthesized nanoparticles, with SEM revealing uniform particle distribution in the composite. Antibacterial activity against *Streptococcus mutans* was assessed using the agar disc diffusion method. Mechanical properties, including flexural strength (FS) and flexural modulus (FM), were evaluated according to ISO 4049 standards. Microhardness was tested according to the American Society for Testing Materials (ASTM E-384:1999) criteria. Polymerization shrinkage was measured using the strain gauge method. Modified composites exhibited significantly greater antibacterial activity against *S. mutans* compared to the control (*p* < *0.001*). The 10 wt.% GSE-TiO₂NPs group demonstrated increased FS and FM values compared to the control group (*p* < *0.05*). All groups surpassed the clinically acceptable microhardness threshold without significant differences (*p* = *0.588*). Polymerization shrinkage was reduced in the modified groups (*p* < *0.01*), with the 20 wt.% GSE-TiO₂NPs group showing the lowest value (13.06 ± 0.92%). Incorporating GSE-TiO₂NPs into composite resins enhances antibacterial activity, improves mechanical properties, and reduces polymerization shrinkage, suggesting a promising approach for developing advanced dental materials with integrated natural bioactive components.

**Graphical abstract:**

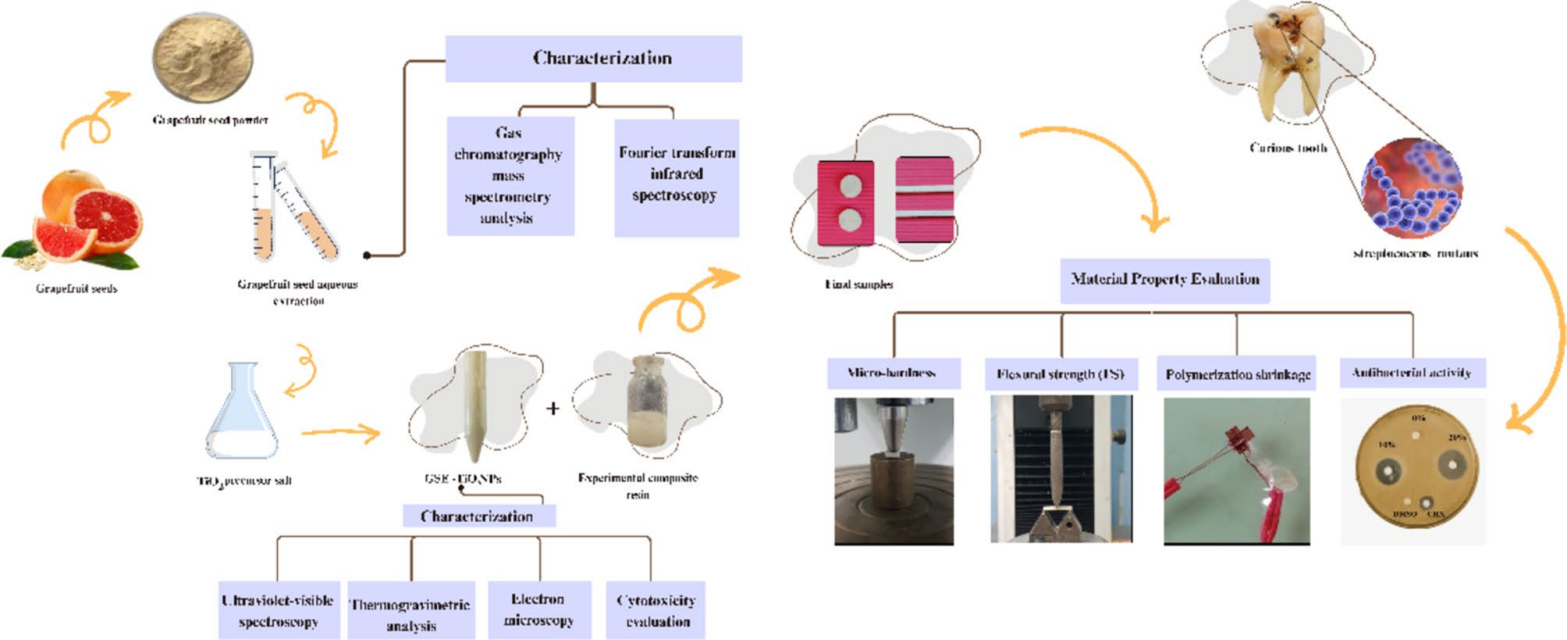

## Introduction

The quest for effective, aesthetically pleasing, and durable restorative materials has driven the development and widespread use of dental composite resins that combine functionality and aesthetics required for successful dental restorations [1]. But dental composite resins still face significant limitations, including susceptibility to secondary caries, shrinking polymerization, and fracture failure [2]. Secondary caries account for 60% of restoration failures in dental practice, causing repetitive replacement of dental restoration that weakens the remaining tooth structure, potentially leading to eventual tooth loss and a significant financial burden on healthcare [3]. In this context, natural products with antibacterial effects have gained significant attention due to their effectiveness and their lack of drug resistance [4]. Also, nanotechnology has revolutionized dental materials by enhancing their mechanical and biological properties [5]. Titanium dioxide nanoparticles are favored for their biocompatibility and antibacterial characteristics, making them suitable for integration into dental composites [5].

Grapefruit seed extract (GSE), *Citrus paradisi*, is well-known for its health benefits and effectiveness against dental caries, dental plaque, and halitosis [6]. Moreover, GSE plays a crucial role in the green synthesis of nanoparticles as a reducing and capping agent [7]. Green synthesis of TiO₂NPs mediated by *Withania* *somnifera* was reported to have broad-spectrum antibiofilm activity against various pathogens [8]. Also, TiO₂NPs mediated by grape seed extract showed excellent antibacterial activity against *Lactobacillus* and *S.mutans* [9]. Modification of composite resin by slilver nanoparticles mediated by *Equisetum sylvaticum* as a filler in 1% wt. and 1.5% wt. enhanced its antibacterial activity against *Streptococcus mutans* without compromising the surface hardness [10].

Despite the proven benefits of GSE and TiO₂NPs individually, there is a paucity of research on their combined application in dental composites. This study pioneers the synthesis of GSE-mediated TiO₂NPs and their incorporation into composite resins, aiming to enhance antibacterial efficacy and mechanical performance. This study aims to bridge this gap by utilizing TiO_2_NPs coated with biomolecules from GSE, which are then used as fillers in experimental composite resin. The null hypothesis assumes that these modifications will not demonstrate antibacterial effects or affect flexure strength, flexure modulus, microhardness, and polymerization shrinkage compared to the control group.

## Materials and methods

### Materials

The materials used in this study included Bisphenol A glycidyl methacrylate (Bis-GMA) with a density of 1.161 g/mL at 25 °C, sourced from Sigma Aldrich (US); Triethylene glycol dimethacrylate (TEGDMA) (99%), containing 200 ppm of monomethyl ether of hydroquinone with a density of 1.092 g/mL at 25 °C; Camphorquinone (CQ) with 97% purity; and 2-(N,N-dimethylamino)ethyl methacrylate (DMAEMA) with 98% purity, containing 700–1000 ppm of monomethyl ether of hydroquinone. Fumed silica nanoparticles and γ-methacryloxypropyltrimethoxysilane with 98% purity were also utilized, along with acetone, and chlorhexidine at a 0.12% concentration. Trypticase Soy Broth (TSB) media was included as the culture medium, while titanium dioxide (CAS 13463-67-7) and dimethyl sulfoxide were supplied by Chem_lab NV (Belgium). *Streptococcus mutans* (ATCC25175) was obtained from the Faculty of Agriculture at Ain Shams University, and grapefruit seeds used in this study were grown and harvested during the spring of 2023 in Nubaria.

### Power analysis

The minimum sample size for this study was 120 specimens. For flexural strength (FS) and flexural modulus (FM) testing, microhardness, antimicrobial testing, and post-gel polymerization shrinkage, 30 specimens were allocated for each test, with 10 specimens per group (Control, 10 wt.% GSE-TiO₂NPs, and 20 wt.% GSE-TiO₂NPs). The sample size was selected based on previous studies (2021, 2023) [10, 11]. The sample size was calculated using the G Power software version 3.1.9.7, with a significance level of 0.05, an effect size of 0.36557, a confidence interval of 95%, and an actual power of 95.23%.

### Extract preparation

The grapefruit seeds were air-dried and processed into powder using an electric blender [12]. Maceration technique was followed to prepare aqueous grapefruit seed extract [13]. Forty grams of seed powder were placed in 160 ml of sterile distilled water at room temperature for 24 h; the crude extract was filtered through Whatman No. 1 filter paper [12]. The extract was stored at 4 °C in a dark place until further use.

### Green synthesis of GSE-TiO₂NPs

For the green synthesis of GSE-TiO_2_NPs, the extract was used as a reducing and capping agent and TiO_2_ as a precursor, the reaction being conducted at room temperature by magnetic stirring according to *Ramya *et al*.* [9]. A solution was prepared by mixing 0.4 g of TiO_2_ with 65 ml of distilled water (DW), then adding freshly prepared GSE to make 100 ml. The reaction mixture was stirred for 10 h and centrifuged at 8000 rpm for 10 min. The pellet was dissolved in DW, concentrated with a rotary evaporator, and vacuum-dried for 24 h to obtain GSE-TiO₂NPs powder. The color change of the reaction mixture was noted, and UV absorbance was measured at regular intervals to confirm the presence of TiO_2_NPs.

### Preparation for experimental composite resin

The resin matrix was prepared by mixing 50 wt.% Bis-GMA and 50 wt.% TEGDMA. The photoinitiators were camphorquinone and DMAEMA at 0.2 wt.% and 0.8 wt.%, respectively. The experimental composite was made by combining 28 wt.% of this matrix with 72 wt.% of fillers [14, 15]. Fillers, either fumed silica or GSE-TiO_2_NPs, were silanized with 5 wt.% (γ-MPTMS). The fillers were uniformly mixed into the resin using a Speed Mixer (Hauschild Speed Mixer DAC 150, FlackTech Inc., Hauschild, Germany) at 3500 rpm for 1 min. The mixture was then placed in a Teflon mold and cured. The specimen grouping was structured as follows:

1. Control group: Unmodified composite containing only fumed silica fillers.
2. 10 wt.% GSE-TiO₂NPs group: Experimental composite containing 10 wt.% GSE-TiO₂NPs.
3. 20 wt.% GSE-TiO₂NPs group: Experimental composite containing 20 wt.% GSE-TiO₂NPs.

### ***Characterization of GSE, GSE-TiO***_***2***_***NPs, and experimental composite resin***

The bioactive components GSE were identified by gas chromatography-mass spectrometry (GC–MS) analysis [16]. Then green synthesis of GSE-TiO_2_NPs was confirmed using visual observation and ultraviolet–visible absorbance spectra (UV‒Vis) [17, 18]. Fourier transform infrared analysis (FT-IR) to identify the main functional groups [19]. Scanning electron microscopy (SEM) was used to examine their morphology, while transmission electron microscopy (TEM) was used to determine their size [20, 21]. For the experimental composite resin, successful preparation was characterized by SEM imaging [22]. X-ray diffraction spectroscopy (XRD) was carried out to observe the crystal structure and phase identification of the synthesized GSE-TiO_2_NPs [23]. Their thermal stability was investigated through thermogravimetric analysis (TGA) [24].

### ***Cytotoxic activity of GSE and GSE-TiO***_***2***_***NPs***

#### Cell culture

Human oral fibroblast (HOrF) cells were cultured in Dulbecco's Modified Eagle Medium (DMEM; Gibco, Thermo Fisher Scientific, USA) supplemented with 10% fetal bovine serum (FBS; Sigma-Aldrich, USA) and 1% penicillin–streptomycin solution (100 U/mL penicillin and 100 µg/mL streptomycin; Invitrogen, USA). Cells were maintained at 37 °C in a humidified atmosphere with 5% CO₂. Cells between passages 3 and 6 were used for all experiments.

#### MTT Assay

HOrF cells were seeded in 96-well plates at a density of 5 × 10^3^ cells per well in 100 µL of complete medium and incubated for 24 h. Cells were then treated with GSE and GSE-TiO₂NPs at concentrations 312.5–10,000 µg/mL. Untreated cells served as the negative control, while cells treated with 0.1% DMSO served as the vehicle control, and 10 µg/mL doxorubicin served as the positive control. Each treatment was performed in triplicate wells. After 24 h of incubation, 20 µL of MTT solution (5 mg/mL in PBS) was added to each well, and the plates were incubated for an additional 4 h at 37 °C. The medium was carefully removed, and 150 µL of DMSO was added to dissolve the formazan crystals. Absorbance was measured at 570 nm using a microplate reader (ELx800, BioTek Instruments, Inc., USA). The viability of the treated cells was calculated as a percentage of the control (untreated cells) using the formula:[25]

$$Viability \left(\%\right)= \frac{Mean OD of control cells}{Mean OD of treated cells} X 100$$

IC₅₀ values were determined using nonlinear regression analysis in GraphPad Prism software (Version 8.0). Data are presented as mean ± SD from three independent experiments.

## Evaluation of experimental composite resin

### Antibacterial activity

#### Minimum inhibitory concentration (MIC)

The minimum inhibitory concentrations (MICs) of GSE and GSE-TiO₂NPs against *Streptococcus mutans* (ATCC25175) from carious dentin were assisted using a standardized microdilution method [26]. Serial dilutions of GSE and GSE-TiO₂NPs were prepared in a 96-well microtiter plate, ranging from 1/2 to 1/64. The MIC, the lowest concentration inhibiting visible bacterial growth, was assessed by measuring optical density (OD) at 600 nm using an ELISA plate reader.

#### Agar diffusion method

The antibacterial activity of GSE, TiO₂NPs, GSE-TiO₂NPs, and the composite resin specimens was evaluated using the agar diffusion method against *S. mutans* [12]*.* TSB agar plates were prepared and dried. The bacterial inoculum was standardized to ~ 1 × 10⁸ CFU/mL and uniformly spread over the agar surface using a sterile cotton swab. Sterile filter paper discs (6 mm diameter) were impregnated with 20 µL of GSE, TiO₂NPs, GSE-TiO₂NPs (equivalent to 2 mg/disc), CHX (0.12% w/v; positive control), or DMSO (10% v/v; negative control). The discs were dried and placed onto the inoculated agar surface. Composite resin specimens (4 mm × 2 mm) containing 0% (control), 10%, and 20% GSE-TiO₂NPs were prepared as previously described, polished, and disinfected. The specimens were placed on separate agar plates inoculated with S. mutans. Plates were incubated at 37 °C for 24 h under anaerobic conditions. Zones of inhibition were measured using a digital caliper in two perpendicular directions, and the mean diameter was calculated. Experiments were conducted in triplicate [12]*.*

#### Flexural strength (FS) and flexural modulus (FM)

Rectangular bar-shaped specimens (2 × 2 × 25 mm) were prepared (*n* = *10*) using a Teflon mold. A three-point bending test was conducted to measure the flexural strength of the specimens using a universal testing machine (INSTRON, 3345 series, Norwood, MA, USA). The specimens were subjected to static loading until fracture occurred, with a crosshead speed of 0.75 mm/min and using a 500 N load cell [27].

#### Vickers microhardness

Disc-shaped composite specimens (6 × 2 mm) were prepared using a split Teflon mold (*n* = *10*). The specimens were finished, and surface microhardness was measured using a Digital Display Vickers Microhardness Tester (Model HVS-50, Laizhou Huayin Testing Instrument Co., Ltd., China). A load of 100 g was applied to the surface of each specimen for 15 s. Three indentations were made on each specimen, and the average was considered [28].

#### Polymerization shrinkage

**Fig. 1:**
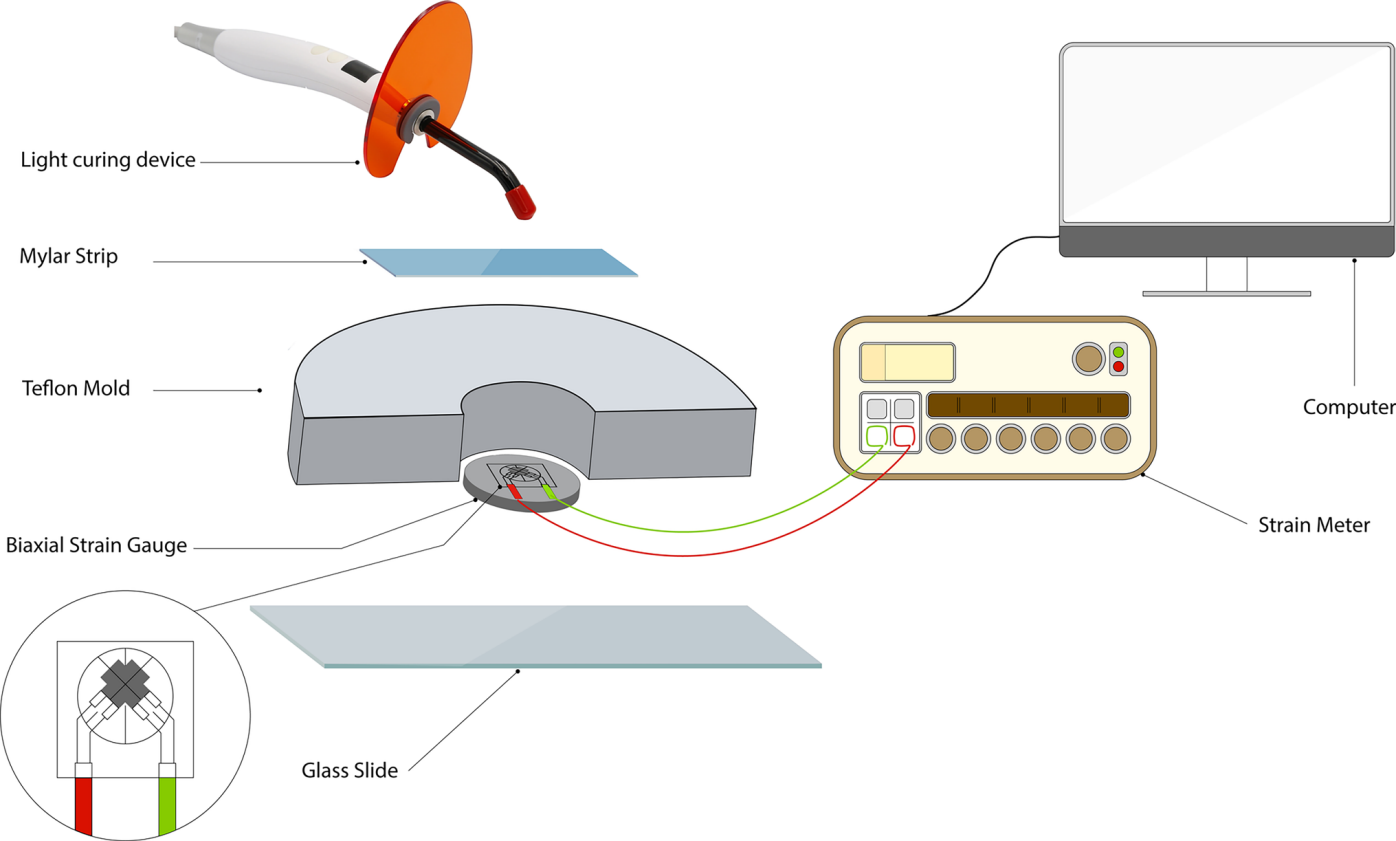
Ischemic illustration showing the experimental design used for measuring post gel shrinkage

Post-gel polymerization shrinkage was measured using the strain gauge method. A foil electrical resistance strain gauge (Kyowa, Ltd., Lot #Y4003S, Japan) was placed in a biaxial configuration and fixed onto a flat glass surface at the center of the mold (4 × 2 mm). The composite was placed into the Teflon mold cavity, centered on the strain gauge. Excess composite material was extruded by applying pressure through a second glass slide, which was then removed. The strain gauge, 1 mm in length with an electric resistance of 119.6 ± 0.4 Ω and a gauge factor of 2.13% ± 1.0%, was connected to a strain monitoring device (Strain Meter, Kyowa, LTD, PCD 300A, Japan) and initially balanced to zero. The strain outputs of both gauges were recorded, and post-gel shrinkage strain was calculated by averaging the two perpendicular strain components, with the time factor held constant at 40 s [29]. Figure 1 shows an ischemic illustration showing the experimental design used for measuring post gel shrinkage.

### Statistical analysis

All data were collected and statistically analyzed using IBM SPSS version 20.0. The Shapiro–Wilk test assessed the normality of distribution. Quantitative data are presented as range (minimum and maximum), mean, and standard deviation. One-way ANOVA tested the significance of antibacterial activity, flexural strength, flexural modulus, microhardness, and polymerization shrinkage at the 5% level, while two-way ANOVA was applied for cytotoxicity results. Pairwise comparisons between groups were performed using Tukey’s post hoc test.

## Results

### Characterization

#### Gas chromatography mass spectrometry) GC–MS)

**Fig. 2:**
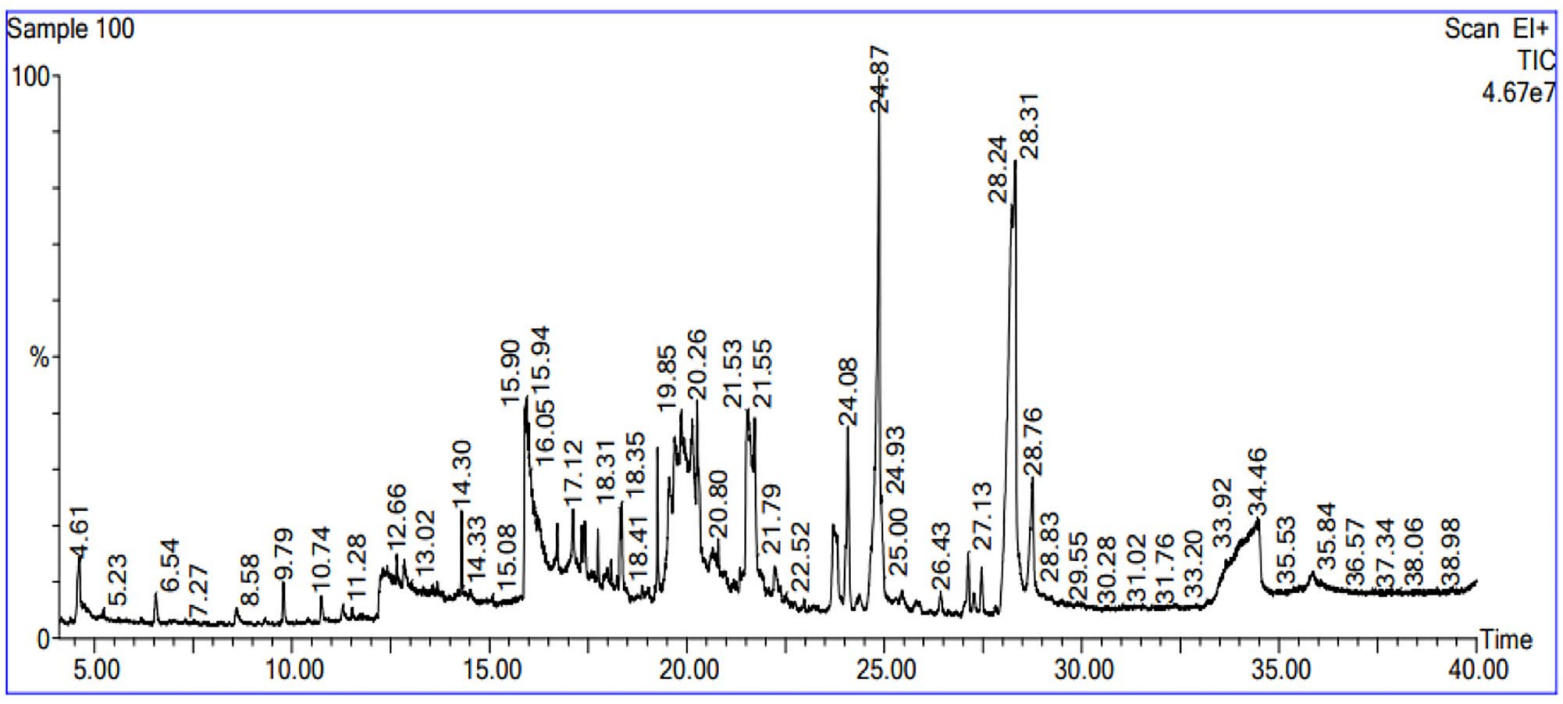
GC–MS spectral chromatogram of grapefruit seed extract

The GC–MS analysis of GSE reveals a rich chemical profile. GC–MS spectral chromatogram of GSE is shown in Fig. 2. The top ten compounds are listed in Table 1. The analysis identified several fatty acids, including nonanoic acid, cis-vaccenic acid, hexanoic acid, palmitic acid, and octadecanoic acid. Additionally, the presence of the neurohormone melatonin was detected. The analysis also revealed terpenoids such as aromadendrene and cubenol.

**Table 1:** Materials used in this study

Components	Suppliers
Bisphenol A glycidyl methacrylate (Bis-GMA) with a density of 1.161 g/mL at 25 °C	Sigma Aldrich, US
Triethylene glycol dimethacrylate (TEGDMA) (99%) contains 200 ppm of monomethyl ether of hydroquinone with a density of 1.092 g/mL at 25 °C.	
Camphorquinone (CQ) (97%)	
2- (N, N-dimethylamino) ethyl methacrylate (DMAEMA) (98%) containing 700–1000 ppm of monomethyl ether of hydroquinone	
Fumed Silica nanoparticles	
γ-methacryloxypropyltrimethoxysilane 98%	
Acetone	
Chlorhexidine (0.12%)	
Trypticase Soy Broth media	
Titanium Dioxide (CAS. 13463-67-7)	
Dimethyl sulfoxide	Chem_lab NV Belgium
*S. mutans* ATCC25175	Faculty of Agriculture, Ain-Shams university
Grapefruit seeds	Grown and harvested during the spring of 2023 in Nubaria

Furthermore, several alcohols, including benzyl alcohol and phenylethyl alcohol, were identified. Other notable compounds detected in the extract included 2-methoxy-4-vinylphenol, 4H-pyran-4-one (2,3-dihydro-3,5-dihydroxy-6-methyl-), and 5-hydroxymethylfurfural. The comprehensive profile highlights the varied chemical composition of grapefruit seed extract and its potential benefits (Table 2).

**Table 2:** The Top Ten Bioactive Components of GSE with Their Peak Area %

	Compound name	Compound structure	RT	Area %
1	Cis-Vaccenic acid		28.318	14.223
2	Palmitic acid		24.872	8.823
3	Melatonin		34.456	7.384
4	7-Tetracyclo [6.2.1.0(3.8)0(3.9)] undecanol, 4,4,11, 11-tetramethyl		19.870	6.059
5	1,2,3-Benzenetriol		15.943	5.556
6	Naphthalene, 1,2,3,4-tetrahydro-1,6-dimethyl-4-(1- methyl ethyl)-, (1S-cis)-		21.550	4.456
7	cis-(-)-2,4a,5,6,9a-Hexahydro-3,5,5,9- tetramethyl(1H) benzocycloheptene		20.140	3.234
8	1-Naphthalenol, 1,2,3,4,4a,7,8,8a-octahedron-1,6- dimethyl-4-(1-methyl ethyl)-, [1R-(1à,4á,4aá,8aá)]-		20.265	2.839
9	1,3-Benzenediol, 4-propyl-		19.560	2.094
10	tau. -Cadinol		19.695	2.091

#### UV–Visible spectrophotometry (UV–Vis)

**Fig. 3:**
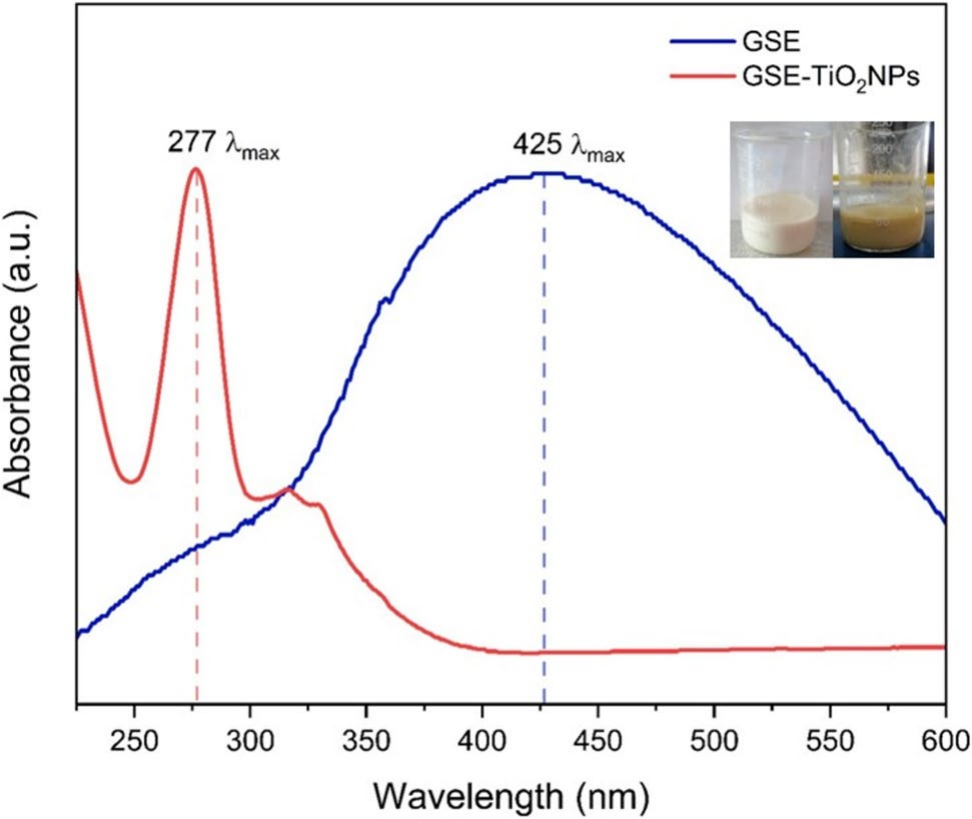
UV–Vis absorption spectra of GSE (Grapefruit Seed Extract) and GSE-TiO₂ NPs

The color of the reaction mixture changed from milk white to pale yellow. The GSE-TiO₂NPs exhibit a distinct absorbance pattern with a significant peak at 275 nm (Fig. 3**)**.

#### Fourier-transform infrared spectroscopy (FTIR)

**Fig. 4:**
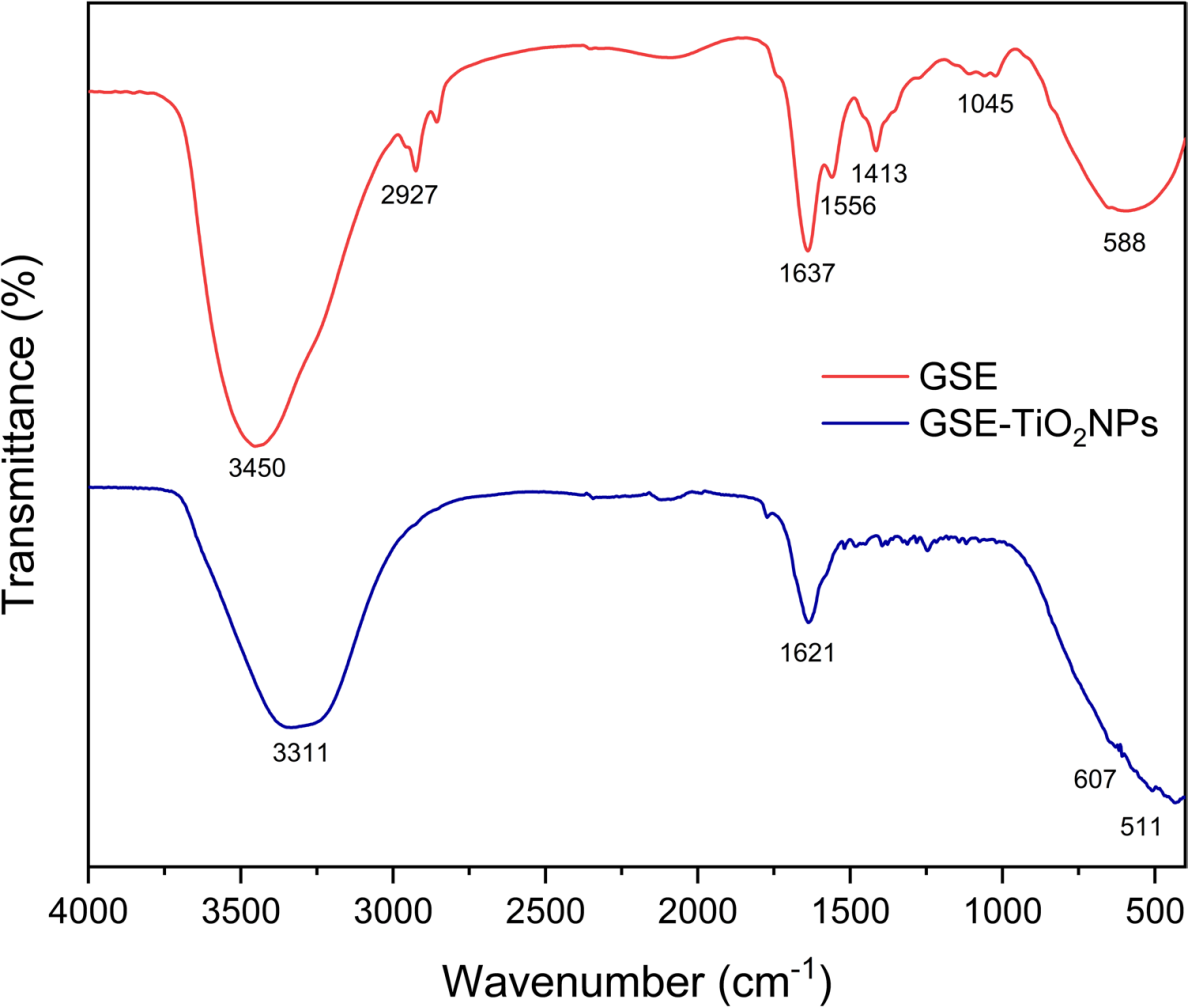
FT-IR analysis of GSE and GSE-TiO_2_NPs

The FT-IR spectrum of GSE displayed a series of characteristic absorption bands, indicative of the various functional groups presented in Fig. 4. The broad absorption band at approximately 3400 cm⁻^1^ indicates the presence of hydroxyl groups (OH), suggesting the presence of alcohols or phenolic compounds. The peaks observed at approximately 2900 cm⁻^1^ correspond to alkyl group (CH) stretching vibrations, indicating the presence of alkyl chains.

The strong absorption band near 1720 cm⁻^1^ suggests the presence of carbonyl groups (C = O), which are characteristic of ketones, aldehydes, or carboxylic acids. The absorption at approximately 1600 cm⁻^1^ can be attributed to the stretching vibrations of carbon–carbon double bonds (C = C), indicating the presence of alkenes or aromatic rings. Complex absorption in the region of 1500–1600 cm⁻^1^ indicates overlapping vibrations of carbonyl and carbon–carbon double bonds, which is indicative of aromatic ketones or conjugated aldehydes.

The peak at approximately 1100 cm⁻^1^ corresponds to ether or ester (C–O) stretching vibrations, suggesting the presence of ether or ester linkages. Absorptions near 750 cm⁻^1^ are indicative of aromatic compound (C–H bending) vibrations, which are commonly associated with out-of-plane bending in aromatic compounds.

While the FT-IR spectrum of GSE-TiO_2_NPs (Fig. 4**)** shows prominent peaks at 1621 cm⁻^1^ and 511 cm⁻^1^ are characteristic of Ti–O–Ti bonds, indicating the presence of titanium dioxide. Additionally, a broad band at 3311 cm⁻^1^ and a peak at 607 cm⁻^1^ suggest the formation of hydroxyl groups and Ti–O–H bonds, respectively.

### Morphological characterization

**Fig. 5:**
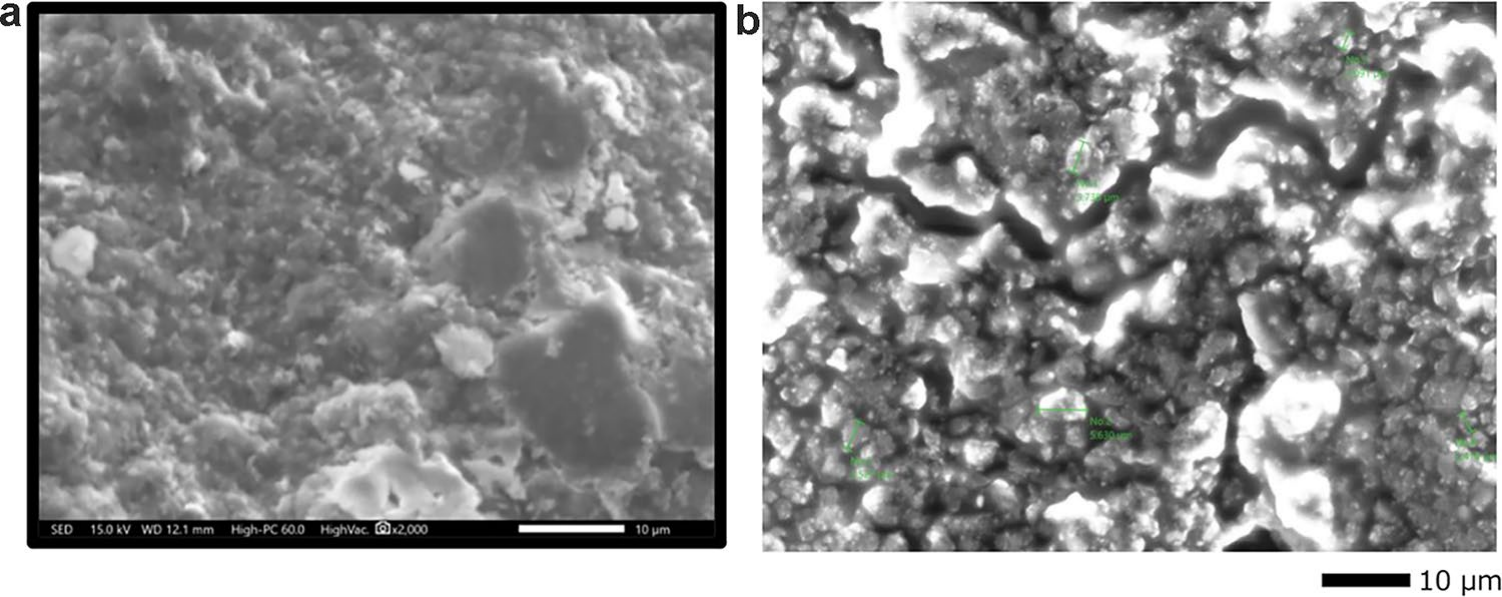
SEM image of (**a**) GSE revealing its heterogeneous nature (**b**) GSE-TiO₂NPs displaying the irregular particle shape

**Fig. 6:**
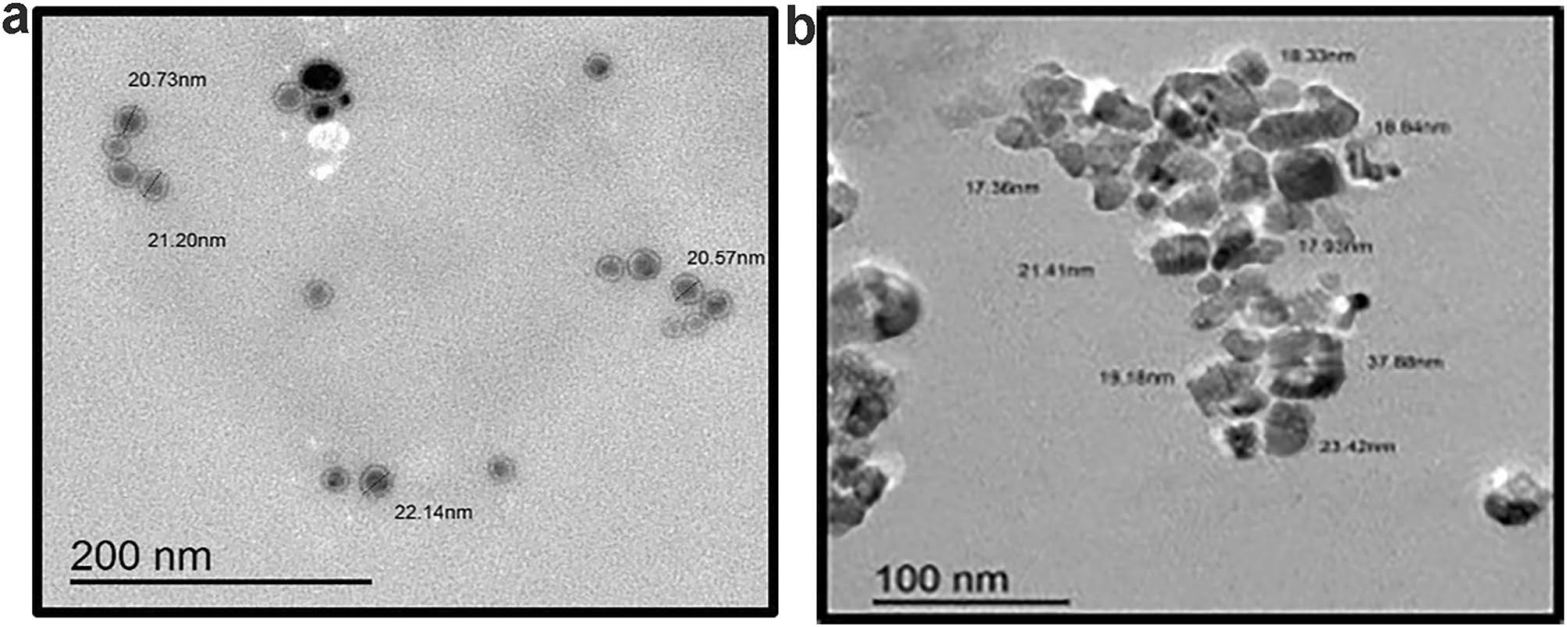
TEM image of (**a**) GSE with uniform shape and size (**b**) GSE-TiO₂NPs showing dispersed polyhedral particles

**Fig. 7:**
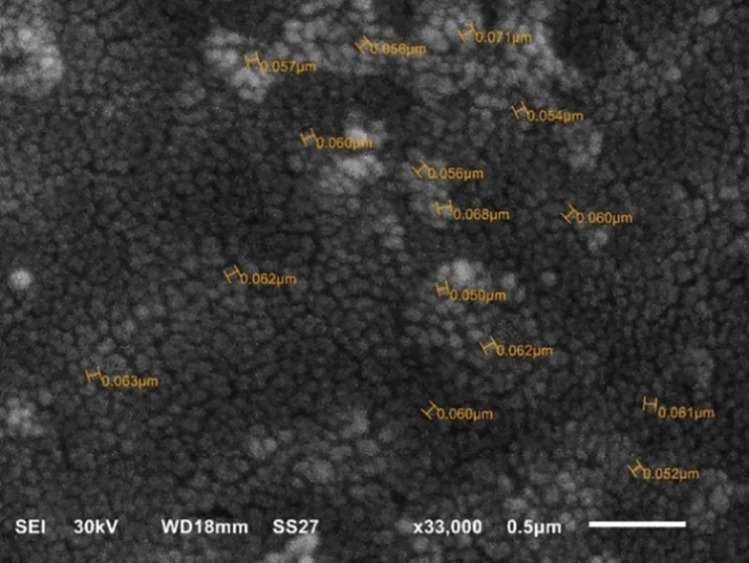
SEM image of the experimental composite resin showing uniformly dispersed nanoparticles within the organic matrix

The SEM images of GSE (Fig. 5a) revealed its irregular, unprocessed state with a diverse mixture of particles. The SEM images of GSE-TiO_2_NPs (Fig. 5b) revealed dispersed TiO_2_NPs of relatively uniform size. TEM analysis of GSE (Fig. 6a) revealed spherical particles with sizes ranging from 20.57 to 22.14 nm. The NPs are relatively uniform in both size and shape, forming small clusters throughout the image. While the GSE-TiO_2_NPs exhibits distinct tetragonal-shaped particles approximately 22.62 ± 4.87 nm in size (Fig. 6b). The SEM images of experimental composite resin showed uniform distribution of spherical nanoparticles and lack of large agglomerates (Fig. 7).

### X-ray diffraction spectroscopy (XRD)

**Fig. 8:**
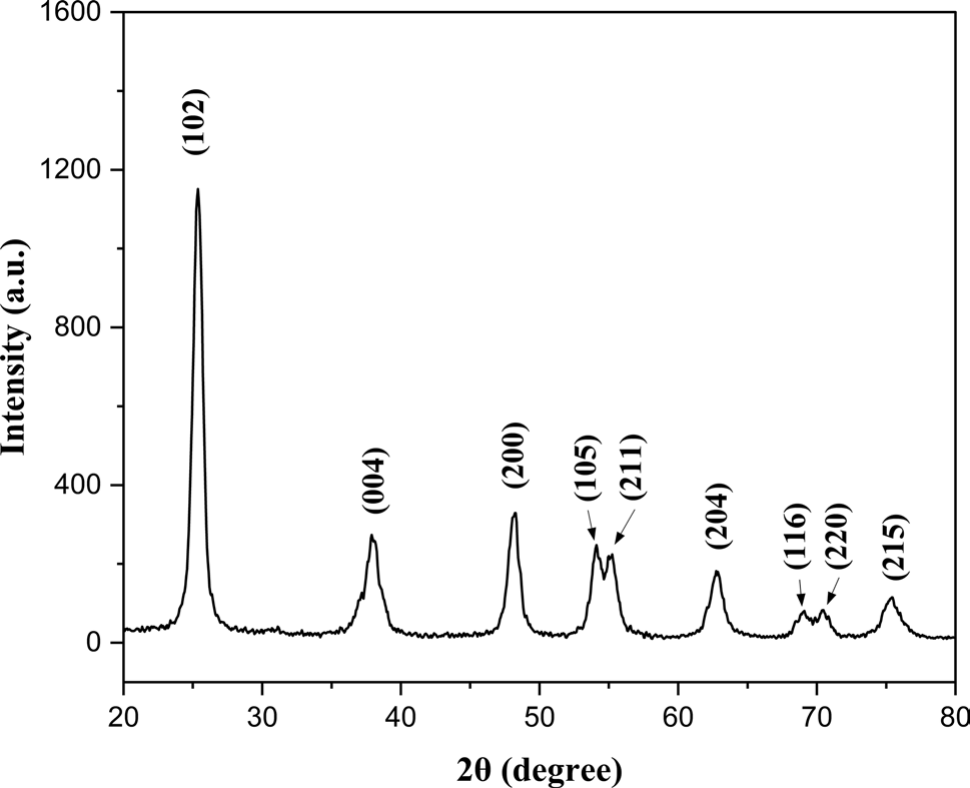
X-ray diffraction pattern of GSE-TiO_2_NPs

The x-ray diffraction spectroscopy of GSE-TiO₂NPs **(**Fig. 8**)** displays diffraction peaks at 25.3°, 37.96°, 48.16°, 54.1°, 55.24°, 62.8°, 68.9°, 70.4°, and 75.46°, which correspond to the planes 101, 004, 200, 105, 211, 204, 116, 220, and 215, respectively, of the tetragonal structure. The 101 crystallographic plane signifies an anatase phase structure of TiO_2_, as referenced in No. 01–084-1285. The size of the anatase particles was calculated to be 19.92 nm.

### Thermogravimetric analysis (TGA)

**Fig. 9:**
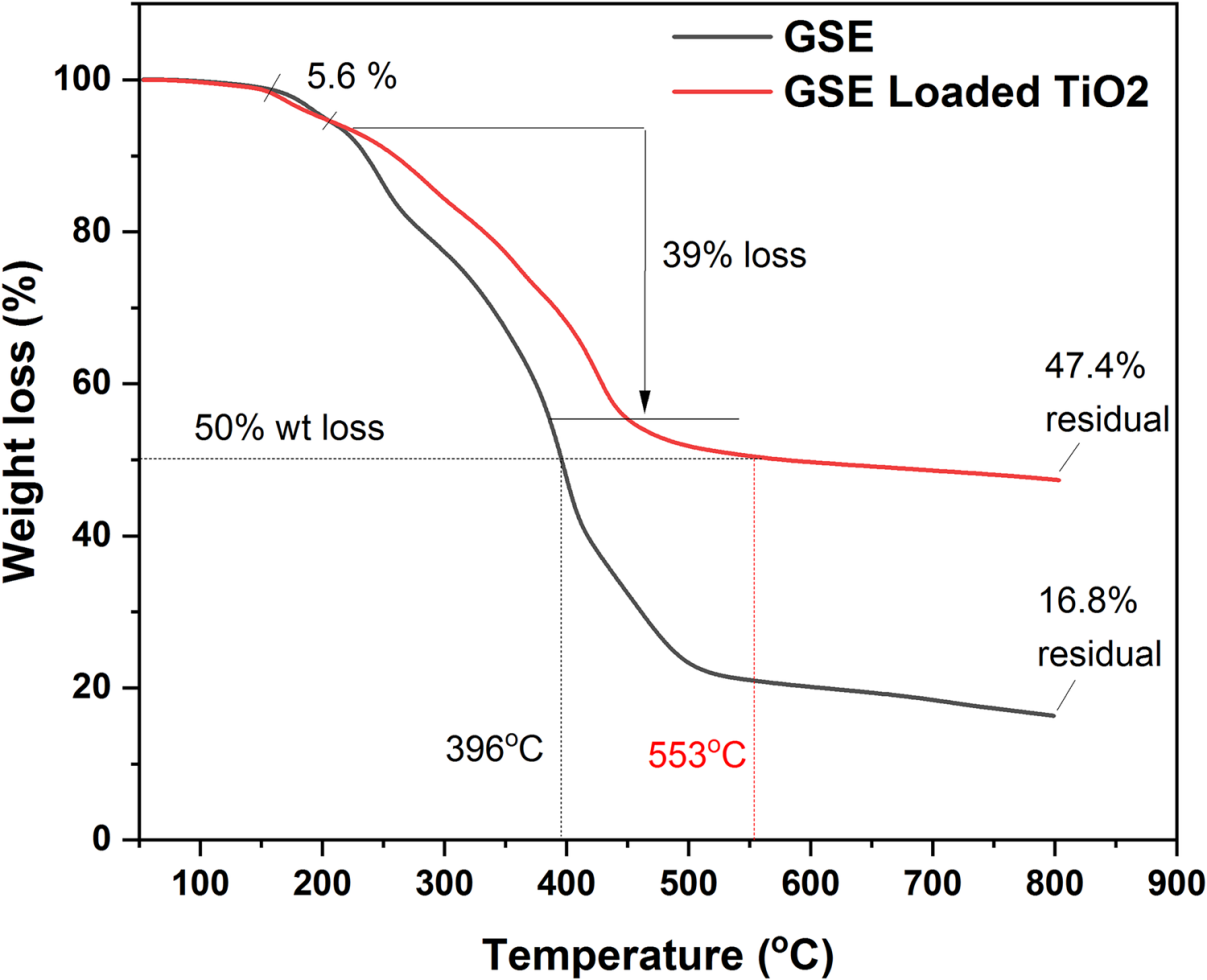
TGA curves of GSE and GSE-TiO_2_NPs

The TGA curve of GSE-TiO₂NPs **(**Fig. 9**)** revealed initial weight loss from room temperature to 150 °C. significant weight loss from 150 to 300 °C, another major weight loss from 300 to 450 °C. GSE shows a similar TGA curve (Fig. 9). However, it exhibited more continuous weight loss, with significant decomposition up to approximately 500 °C, reflecting the thermal degradation. This highlights the enhanced thermal stability of GSE-TiO₂NPs compared with GSE.

### ***Cytotoxic activity of GSE and GSE-TiO***_***2***_***NPs***

#### Cytotoxicity

The findings reveal that GSE and GSE-TiO_2_NPs exhibit marked concentration-dependent cytotoxicity, reducing cell viability significantly at higher concentrations. At 10,000 μg/mL, GSE showed 79.26% toxicity, whereas at lower concentrations (312.5 μg/mL), it displayed no significant toxicity. Also, GSE-TiO_2_NPs at 10,000 μg/mL showed 70.38% toxicity, whereas at lower concentrations (312.5 μg/mL), it displayed no significant toxicity. The IC_50_ values indicate the concentration at which 50% cell viability is inhibited. GSE had an IC_50_ of 332.615 μg/mL, while GSE-TiO_2_NPs had an IC_50_ of 502.598 μg/mL.

**Fig. 10:**
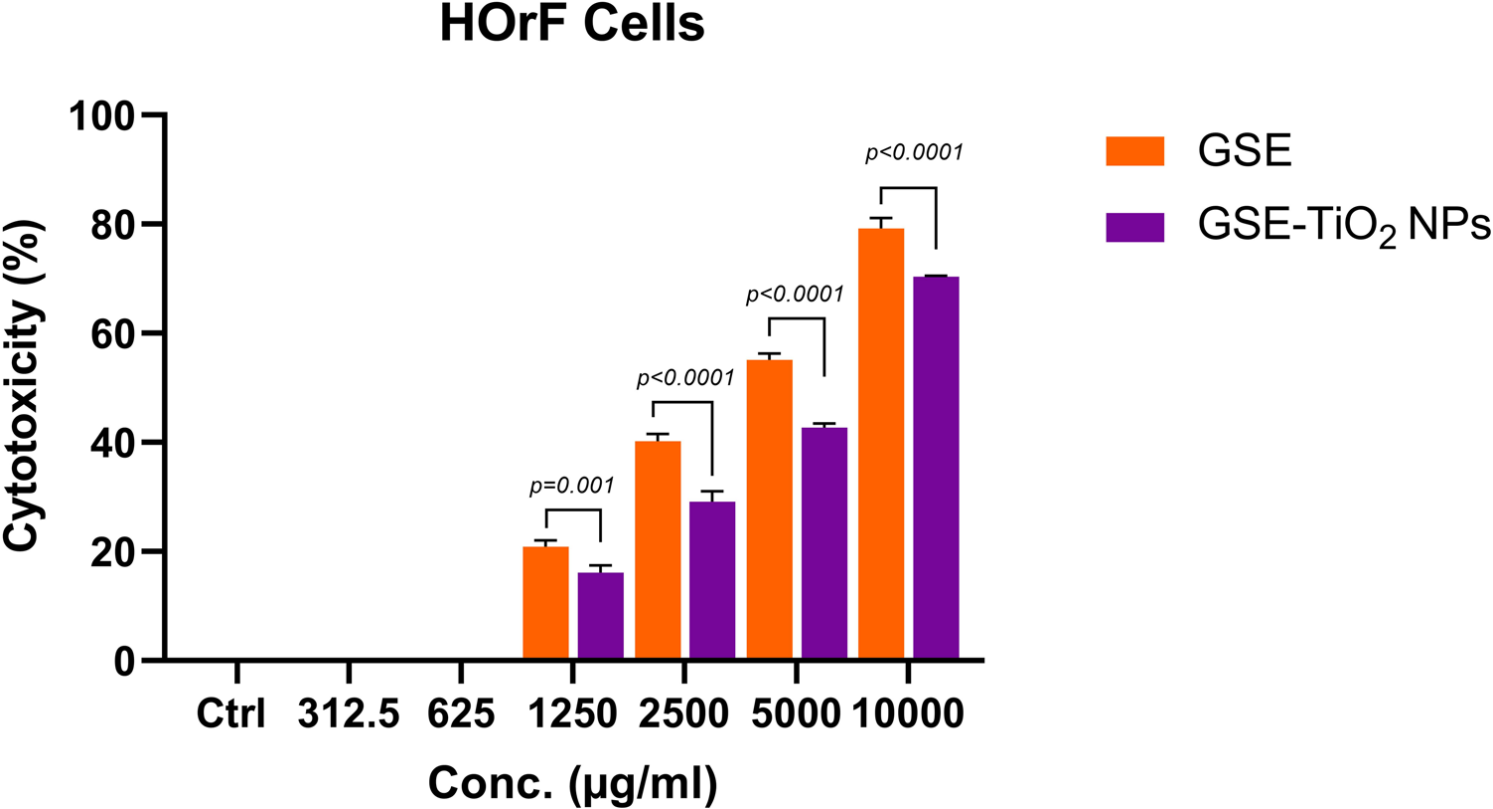
Cytotoxicity of GSE and GSE-TiO_2_NPs

A two-way ANOVA revealed a very highly significant difference in cytotoxicity between GSE and GSE-TiO₂NPs (*P* < *0.0001*) across all concentrations (Fig. 10). This suggests that GSE-TiO₂NPs are significantly less toxic than GSE alone, particularly at lower concentrations. The reduced cytotoxicity of GSE-TiO₂NPs makes it a more favorable candidate for biomedical applications where reduced toxicity is crucial.

**Fig. 11:**
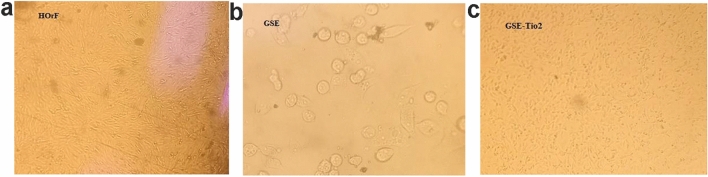
**A** Untreated HOrF Cells. **B** Morphological Changes in HOrF Cells Treated with GSE. **C** Morphological Changes in HOrF Cells Treated with GSE-TiO_2_NPs

The light microscopy images in Fig. 11 further illustrate the cytotoxic effects compared to untreated HorF (Fig. 11A). HOrF cells treated with 10,000 μg/mL of GSE show significant morphological changes such as cell rounding and detachment, indicating increased cytotoxicity (Fig. 11B). In contrast, Fig. 11C demonstrates fewer morphological changes in cells treated with GSE-TiO₂NPs at the same concentration, reinforcing the observation of lower cytotoxicity. The images clearly show that GSE-TiO₂NP-treated cells maintain a more intact cellular structure, suggesting that the incorporation of GSE-TiO₂NP effectively reduces the cytotoxic impact of GSE alone.

#### Antibacterial activity

**Fig. 12:**
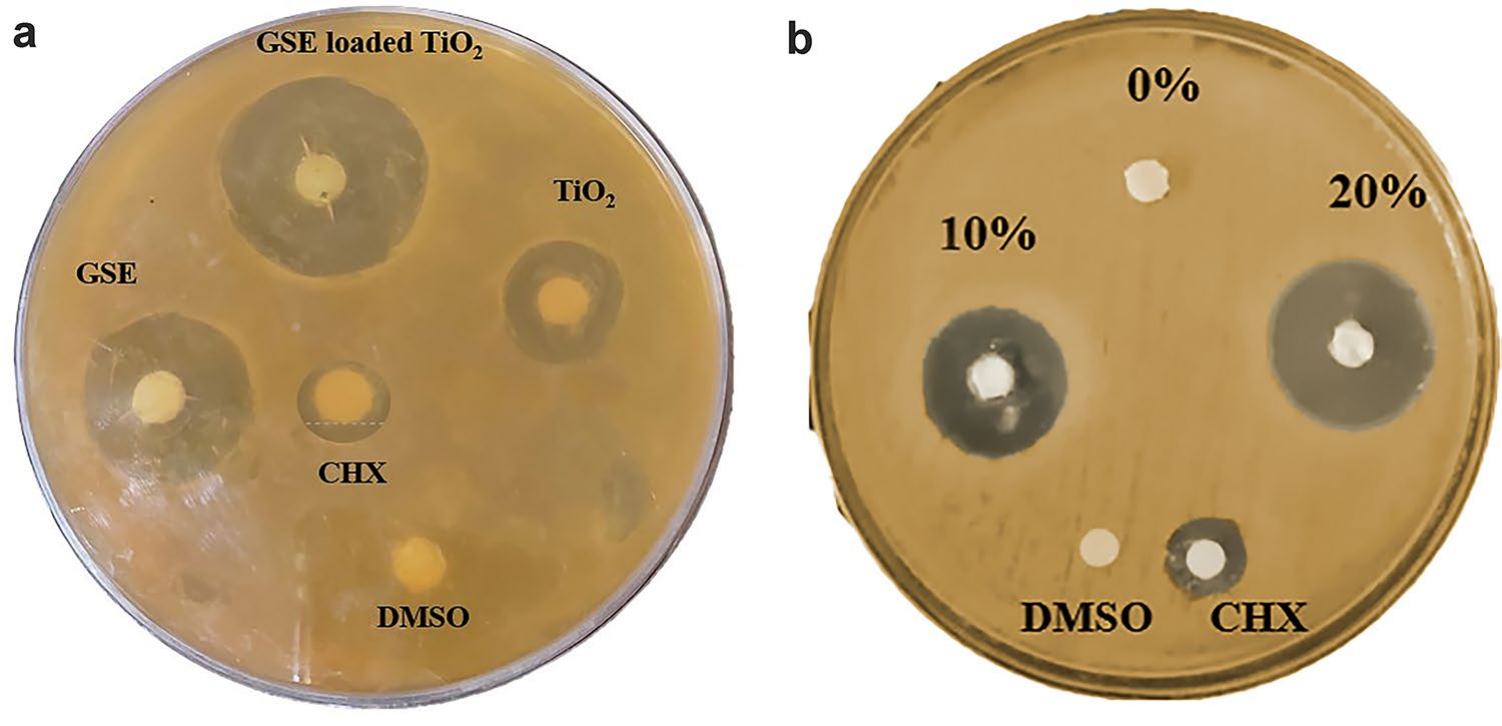
Agar diffusion test of (**A**) GSE, TiO₂NPs, GSE-TiO₂NPs, chlorhexidine, and DMSO (**B**) unmodified composite (0%), composite with 10% and 20% GSE-TiO₂NPs, composite with 20% GSE-TiO₂NPs

The highest antibacterial activity was observed for GSE-TiO₂NPs prior to incorporation into the composite resin, with a mean inhibition zone of 32.6 ± 5.81 mm (Fig. 12A**)**. Upon incorporation into composite resin, GSE-TiO₂NPs showed reduced antibacterial effect compared to their free form (Fig. 12B). For the modified experimental composite, 20% GSE-TiO₂NP composite exhibited the highest antibacterial activity (25.60 ± 1.14 mm), followed by 10% GSE-TiO₂NP composite (20.0 ± 1.58 mm).

**Fig. 13:**
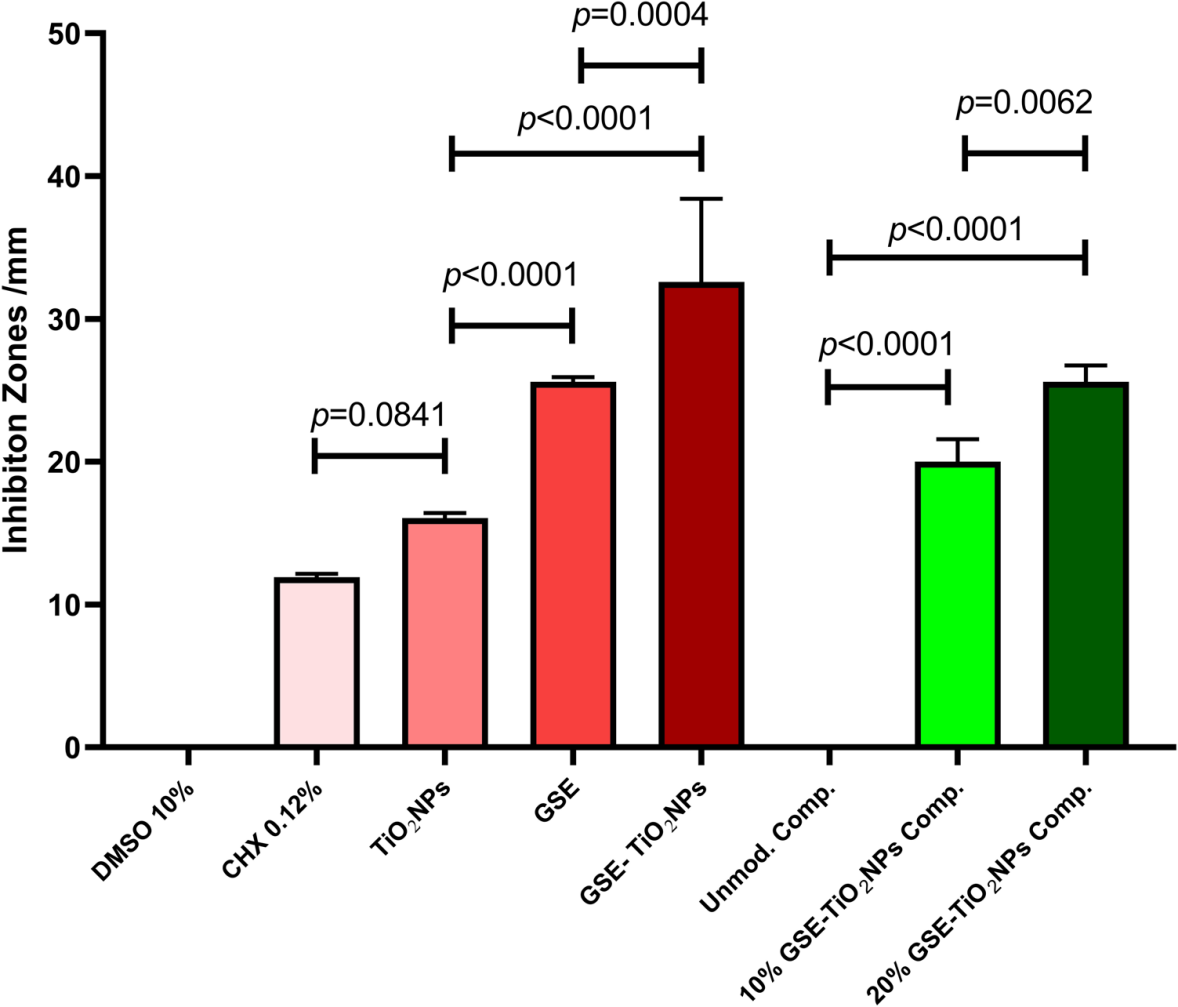
antibacterial activity means (inhibition zone in mm) of all tested groups

There was an extremely significant difference between all tested compounds (*P* < *0. 00001*). GSE-TiO₂NPs showed an extremely highly significant effect than GSE and a very highly significant antibacterial activity than TiO_2_NPs alone (Fig. 13**)**.

For experimental composite resin, the 20 wt.% GSE-TiO₂NPs group demonstrated extremely highly significant antibacterial activity compared to the control and the 10 wt.% GSE-TiO₂NPs group (Fig. 13**)**.

#### Flexural strength (FS) and modulus (FM)

The highest Flexural Strength (FS) mean values were observed in the 10 wt.% GSE-TiO₂NPs group (91.22 ± 3.15) MPa. There was a statistically significant difference between all the tested groups (*P* = 0.043) (Table 3**)**. The highest mean values of FM were in the 10 wt.% GSE-TiO₂NPs group (7.73 ± 0.27) GPa (Table 3**)**.

**Table 3:** One-Way ANOVA results of flexural strength (MPa), flexural modulus (GPa), microhardness (VHN), and post gel polymerization shrinkage

Tested properties	Group I	Group II	Group III	*P*-Value
	Mean ± SD	Mean ± SD	Mean ± SD	
Flexural strength (MPa)	80.11 ± 10.03^a^	91.22 ± 3.15^bc^	83.28 ± 2.86^ac^	0.043*
Flexural modulus (GPa)	6.79 ± 0.85^a^	7.73 ± 0.27^bc^	7.0 ± 0.29^ac^	0.043*
Microhardness (VHN)	80.38 ± 2.85^a^	81.31 ± 1.99^a^	81.77 ± 1.25^a^	0.588
Post gel polymerization shrinkage	32.41 ± 3.98^a^	18.63 ± 2.63^b^	13.06 ± 0.92^c^	< 0.001*

In the same row, values with the same lowercase letters have no significant difference

*Statistically significant at *P* < 0.05 after post hoc (Tukey) test

#### Vickers microhardness

The highest Vickers Microhardness (VHN) mean values were recorded in the 20 wt.% GSE-TiO₂NPs group (81.77 ± 1.25) VHN. There was no statistically significant difference among the tested groups (*P* = 0.588).

#### Polymerization shrinkage

The lowest polymerization shrinkage strain mean values were recorded in the 20 wt.% GSE-TiO₂NPs group (13.06 ± 0.92). There was a highly significant difference in polymerization shrinkage amongall tested groups (*P* < 0.001) (Table 3**)**. Both modified groups were highly significantly lower than control (*P* = *0.001*).

In the same row, values with the same lowercase letters have no significant difference. *Statistically significant at *P* < 0.05 after post hoc (Tukey) test.

## Discussion

The GC–MS analysis proved the rich chemical profile of GSE with diverse bioactive components. The analysis identified several fatty acids, including nonanoic acid, cis-vaccenic acid, hexanoic acid, palmitic acid, and octadecanoic acid. Additionally, the presence of the neurohormone melatonin was detected. The analysis also revealed terpenoids such as aromadendrene and cubenol. Furthermore, several alcohols, including benzyl alcohol and phenylethyl alcohol, were identified. Other notable compounds detected in the extract included 2-methoxy-4-vinylphenol, 4H-pyran-4-one (2,3-dihydro-3,5-dihydroxy-6-methyl-), and 5-hydroxymethylfurfural. This comprehensive profile highlights the complex chemical composition of GSE and its potential applications as a stabilizing and reducing agent. Also are responsible for its antimicrobial activity [30–32].

Successful green synthesis of GSE-TiO_2_NPs was confirmed by the color change of the reaction mixture from pale yellow to milk white. This finding is consistent with the work of *Farook and Ramasamy *et al*.* [33, 34]*,* who synthesized titanium nanoparticles using *Citrus lemon* and *Ludwigia octovalvis*, respectively. The observed color change can be attributed to the presence of alkaloids, flavonoids, and polyphenols in GSE, which act as reducing agents for metal ions [7] While visual observation is common in green synthesis as an initial indicator, further confirmation was provided in this study through UV–Vis spectroscopy, FT-IR spectroscopy, and TEM analysis. The occurrence of strong absorption peak at 275 nm indicated the formation of TiO_2_NPs in the reaction mixture. This observation aligns with the findings of studies conducted by *Rajput* and *Rathi *et al., which reported similar peaks for TiO_2_NPs [35, 36].

FT-IR is a sensitive, rapid, selective, and authentic analytical technique used to identify the functional groups of plant samples [37]. FT-IR analysis of GSE reflected functional groups related to alcohols, phenolic compounds, ketones, aldehydes, and acids. For GSE-TiO_2_NPs, the presence of Ti–O–Ti and Ti–OH stretching peaks in the GSE-TiO_2_NPs supports the successful green synthesis process. The similarity of O–H stretching peaks in both spectra indicates that hydroxyl groups from the GSE adsorb onto the TiO₂NPs highlighting the role of GSE in the green synthesis process [35, 36].

The diverse mixture of particles in SEM images of GSE, is common in plant-based materials containing a variety of compounds and fibrous materials, such as phenolics, tannins, and other bioactive components [38]. The aggregation observed may occur due to the natural adhesive properties of the phenolic compounds present in the extract [39]. The SEM image of GSE-TiO_2_NPs, Isolated particles were observed, indicating that the effectiveness of GSE as a capping agent preventing nanoparticles from clumping together.

TEM analysis showed the uniform size and shape of the GSE-TiO_2_NPs suggests a high level of control in the synthesis process as a reducing agent. This uniformity is crucial as it can influence the mechanical properties and the efficacy of the material. SEM and TEM findings align with a study by *Bahari *et al*.* (2023) [40], that explored the use of honey in green synthesis of silver and zinc oxide nanoparticles highlighting the dual role of honey as both a reducing and capping agent. The successful synthesis of experimental composite resin was confirmed by the uniform particle distribution, lack of large agglomerates and appropriate size range observed in the SEM image [22].

The crystallite size of the nano particles was calculated using Scherrer equation [41–43]. The findings of XRD correspond with earlier studies, indicating a crystallite size close to the previously reported value by *Li *et al. and *Saravanan *et al. [23, 44]. The TGA curve of GSE-TiO₂NPs revealed initial weight loss from room temperature to 150 °C due to evaporation of adsorbed water and volatiles. These findings suggest its suitability for use in the oral cavity that experiences temperature fluctuations around 70 °C [45]. From 150 to 300 °C, significant weight loss indicates decomposition of organic compounds and residual reactants from synthesis. Another major weight loss from 300 to 450 °C suggested further decomposition of the organic materials. Above 450 °C, the weight stabilized, indicating that the pure titanium dioxide nanoparticles were thermally stable. GSE shows similar TGA curve. However, it exhibited more continuous weight loss, with significant decomposition up to approximately 500 °C, reflecting the thermal degradation. This highlights the enhanced thermal stability of GSE-TiO₂NPs compared with GSE [46].

GSE-TiO₂NPs were significantly less toxic than GSE, particularly at lower concentrations. In this study, The IC_50_ values were 333 μg/mL for GSE and 503 μg/mL for GSE-TiO_2_NPs. Additionally, MICs values were 40 μg/mL for GSE and 200 μg/mL for GSE-TiO_2_NPs, both of which are below their respective IC_50_ values, ensuring effective antimicrobial action at safe concentrations. GSE-TiO₂NPs showed extremely highly significant antimicrobial effect than either GSE or TiO_2_NPs alone. This indicates a potent synergistic antimicrobial effect. For GSE, the extraction was freshly performed using to avoid potential confounding antimicrobial effects from preservatives like benzalkonium and benzethonium compounds commonly found in commercially available products. This ensured that any observed antimicrobial effect was solely attributable to the extract itself.

Upon incorporation into composite resin, GSE-TiO₂NPs showed reduced antimicrobial effect compared to their free form. These findings were supported by *mona *et al*.* [47] revealing that zinc oxide nanoparticles showed significant antibacterial activity when directly tested. But their incorporation into composite resin resulted in a diminished effect due to potential binding or encapsulation within the resin matrix, which restricts their availability to exert antimicrobial action. For modified experimental composite, composite with 20% GSE-TiO₂NP exhibited the highest antimicrobial activity. So, the null hypothesis was rejected regarding the antimicrobial effect. The potential explanation for the enhanced antimicrobial activity of GSE-TiO_2_NPs may be the sum of the antibacterial effectiveness of GSE, TiO₂NPs and the synergistic effect between them both arising from the green synthesis approach.

For GSE, the observed antimicrobial activity can be attributed to the bioactive compounds detected through GC–MS and FT-IR analysis. Such as Cis-Vaccenic acid is a kind of trans-fatty acid known for its antibacterial activity against different pathogens [48]. The hexadecenoic acid, often known as palmitic acid, was detected. It is a promising antibacterial action against both Gram-positive and Gram-negative bacteria [49]. Melatonin (*N*-acetyl-5-methoxytryptamine) has been considered a significant antibiotic, anti-parasitic, and antiviral [47]. 7-Tetracyclo [6.2.1.0(3.8)0(3.9)] undecanol, 4,4,11, 11-tetramethyl is effective against *Pseudomonas sp*, *Staphylococcus aureus,* and *Aspergillus flavus* [50]. (1,2,3-benzene-triol), Pyrogallic acid, is a functional polyphenolic compound with antimicrobial and antioxidant capacities [51]. Also, FT-IR analysis of GSE reflected many important compounds with antimicrobial activities such as alcohols, phenolic compounds, ketones, aldehydes, and acids.

Titanium nanoparticles reduce bacterial colonization and inhibit biofilm formation. Besides, the photocatalytic activity of TiO₂NPs on exposure to light generates reactive oxygen species that damage bacterial cells and inhibit their growth [52]. *Sodagar *et al*.* (2017) [53] demonstrated that composites containing 10% TiO₂NPs exhibited inhibition zones of 11.33 ± 1.5 mm. However, the present study reveals a greater antimicrobial efficacy with composites containing 10% GSE-TiO₂NPs, which showed inhibition zones of 20.0 ± 1.58 mm likely due to the synergistic effects of the bioactive compounds in GSE, such as flavonoids and phenolic acids, which possess intrinsic antimicrobial properties [9]. GSE not only stabilizes the TiO₂NPs but also augments their antimicrobial effectiveness beyond that observed with chemically synthesized TiO₂NPs.

Enhancing the antimicrobial capability of composites may adversely affect their mechanical properties. Therefore, the mechanical properties were evaluated. Flexural strength (FS) is frequently examined due to its greater sensitivity to subtle changes in the material substructure [54]. The flexural modulus (FM) characterizes stiffness with higher values indicating greater stiffness [55]. The FS values of all the tested groups exceeded 80 MPa, which is consistent with the ISO reference [56]. These acceptable values may be attributed to the fact that fillers appeared as spherical nanoparticles uniformly distributed in the resin matrix in the SEM images. This is expected to minimize stress concentration and enhance the load distribution. Also, spherical nanofillers can accommodate increased filler loads in composites [43]. The highest FS and FM values were recorded for the composite containing 10 wt.% GSE-TiO_2_NPs.These observations could be attributed to the uniform distribution of extremely small GSE-TiO_2_NPs within the composite, enabling strong particle–matrix interfacial bonding and strong load transfer leading to increased flexural strength and modulus [5, 57]. So, the null hypothesis was rejected by FS and FM.

The null hypothesis was rejected for the microhardness as there was no significant difference between all tested groups. The Vickers microhardness values of all tested groups ranged from 75 to 82 HV, which is twice the acceptable range provided by ISO 4049 (40 HV) [56]. This notable increase can be attributed to the inclusion of fumed silica in all the groups. Previous studies have reported that silica NPs can significantly increase the hardness of modified nanocomposites compared to unmodified specimens [58]. For instance, composites with silica NPs improved surface hardness by 24 times compared to unfilled resin [59]^.^ The uniform dispersion of nanoparticles within the resin matrix further contributes to this improvement [60].

The results of this study are in agreement with *Azmy *et al*.* [5] who explored the impact of different nanoparticles, such as TiO₂NPs, on the FS of composite resins and found that the incorporation of TiO₂NPs, significantly enhanced the FS at lower concentrations (3 wt.%) and explained the observed results owing to that the nanoparticles facilitated load redistribution, leading to improved mechanical properties. While (7 wt.%) significantly reduced the flexural strength due to agglomeration of TiO_2_NPs. In disagreement, the incorporation of 10% GSE-TiO₂NPs in this study significantly enhanced the flexural strength (FS) and flexural modulus (FM) of composite resins. While the 20% concentration didn’t compromise these properties. This highlights the role of GSE as a capping agent preventing agglomeration. Polymerization stresses can be categorized into pre gel and post gel stages. During the pre-gel stage, stress can be relieved by material flow.

However, in the post gel stage, material flow stops, preventing stress relaxation. Consequently, post gel stress persists and is clinically significant. In this study, Polymerization shrinkage (PS) was evaluated using strain gauge method, which isolates the clinically significant portion of total volumetric shrinkage and allows precise localized measurement and provides real-time monitoring of shrinkage during curing [29]. A significant reduction in post-gel (PS) was recorded with the incorporation of GSE-TiO_2_NPs into composite resin. So, the null hypothesis was rejected. This improvement is attributed to the uniform distribution of small GSE-TiO_2_NPs, which effectively seal spaces between polymer matrix chains and limit the segmental movement of macromolecular chains, thereby reducing polymerization [61].

The findings of this study demonstrate the potential of GSE-TiO₂NPs to significantly enhance the antimicrobial, mechanical and physical properties of dental composites. This novel approach not only addresses the common issues of secondary caries sand polymerization shrinkage but also introduces an eco-friendly, sustainable method for improving dental materials. However, this study was conducted in vitro. Consequently, more research is needed to assess the material’s performance under conditions closely resemble the oral environment. Future studies should investigate the effect of GSE-TiO₂NPs on the color stability of composite resins.

## Limitations and future directions

While the in vitro results are promising, they may not fully replicate the complex conditions of the oral environment. Factors such as saliva composition, enzymatic activity, temperature fluctuations, and mechanical stresses could influence the performance of GSE-TiO₂NPs-enhanced composites in vivo. Therefore, further studies are warranted to evaluate the long-term durability, biocompatibility, and clinical efficacy of these composites under simulated oral conditions or through in vivo experiments. Additionally, future research should investigate the potential effects of GSE-TiO₂NPs on the color stability and esthetic properties of composite resins, as well as their interaction with other restorative materials. Exploring different concentrations and combinations of nanoparticles could optimize the balance between antibacterial efficacy and mechanical performance.

## Conclusion

This study demonstrates that the incorporation of GSE-TiO₂NPs into dental composite resins significantly enhances antibacterial activity against *Streptococcus mutans* while improving mechanical properties such as flexural strength and flexural modulus and reducing polymerization shrinkage without compromising microhardness. The synergistic effect of bioactive compounds from GSE and the photocatalytic properties of TiO₂NPs offers a novel approach to developing advanced dental composites that address common clinical challenges. These findings contribute to the advancement of dental materials science and hold potential for translating into improved restorative practices.

## Data Availability

The data used to support the findings of this study are included within the article.

## Abbreviations

BIS-GMA: Bisphenol A-glycidyl dimethacrylate
TEGDMA: Triethylene glycol dimethacrylate
TIO_2_NPs: Titanium dioxide nanoparticles
MPTMS: Y-Methacryloxypropyltrimethoxysilane
CQ: Camphorquinone (4,7,7-trimethylbicycloheptane-2,3-Dione)
DMAEMA: 2- (N, N-dimethylamine)-ethyl methacrylate
ASTM: American society for testing materials
ISO: International organization for standardization
